# The acute effects of caffeine, creatine, and carbohydrate mouth rinse combined with conditioning activity on subsequent sports performance: a systematic review and Bayesian meta-analysis

**DOI:** 10.3389/fnut.2025.1669004

**Published:** 2025-11-06

**Authors:** Yanfeng Li, Bopeng Qiu, Hongyan Kuai, Kongyun Huang, Hengxian Liu, Mingyang Zhang, Mingyue Yin, Yilin Zhang, Hengzhi Deng, Yuming Zhong, Hao Kong, Jun Chen, Kai Xu

**Affiliations:** 1 Basketball Instructional Department, Institute of Physical Education and Training, Capital University of Physical Education and Sports, Beijing, China; 2 School of Strength and Conditioning, Beijing Sport University, Beijing, China; 3 Division of Sports Science & Physical Education, Tsinghua University, Beijing, China; 4 School of Athletic Performance, Shanghai University of Sport, Shanghai, China; 5 Complex Systems Group & GISC, Universidad Rey Juan Carlos, Móstoles, Spain; 6 School of Sport Science, Jishou University, Jishou, China; 7 The Mary MacKillop Institute for Health Research, Australian Catholic University, Melbourne, VIC, Australia; 8 Athletic Training Institute, Tianjin University of Sport, Tianjin, China; 9 Faculty of Sports and Exercise Science, University Malaya, Kuala Lumpur, Malaysia; 10 School of Medical and Health Sciences, Edith Cowan University, Joondalup, WA, Australia

**Keywords:** post-activation performance enhancement, caffeine, creatine, carbohydrate mouth rinse, ergogenic aid

## Abstract

**Background:**

Post-activation performance enhancement (PAPE) refers to the acute improvement in sports performance following a conditioning activity (CA), commonly used in warm-up protocols and complex training. While CA alone has demonstrated performance benefits, the effects of combining CA-induced PAPE with supplements (e.g., caffeine, creatine, or carbohydrate mouth rinse) remain unclear.

**Objectives:**

This study was to (i) assess the effects of PAPE combined with supplements on sports performance and fatigue resistance compared to PAPE + placebo, warm-up + supplements, or warm-up alone, and (ii) synthesize direct and indirect evidence on performance outcomes using network meta-analysis.

**Methods:**

Searches were conducted across three databases. Eligible studies were randomized controlled or crossover trials involving recreationally active individuals, comparing PAPE combined with supplements to interventions (i.e., PAPE + placebo, warm-up + supplements, or warm-up alone). Outcomes related to sports performance or fatigue were analyzed using a multilevel Bayesian approach incorporating pairwise and multiple (network) comparisons.

**Results:**

Ten studies involving 198 participants were included. Current evidence indicates that the probabilities of positive effects (effect size > 0) on sports performance for PAPE combined with supplements compared to PAPE + placebo, warm-up + supplements, warm-up + placebo, and warm-up alone were 90.83, 85.09, 92.29, and 88.10%, respectively. Additionally, PAPE combined with supplements showed an 83.65% probability of superior fatigue resistance compared to PAPE + placebo. Subgroup analysis indicated that plyometric CA (i.e., jump and sprint) combined with supplementation (all were caffeine) was more effective than plyometric CA + PLA (ES = 0.97, >0 probability = 99.79%). Network meta-analysis identified PAPE + caffeine (SURCA = 83.40%) and PAPE + carbohydrate mouth rinse (SURCA = 78.40%) as the most effective interventions for enhancing sports performance, with PAPE + caffeine exhibiting a 99.17% probability of positive effect compared to warm-up alone.

**Conclusion:**

Preliminary evidence suggests that combining caffeine with plyometric CA is the most effective strategy for enhancing sports performance. Although creatine and carbohydrate supplementation alongside CA may provide some benefits, their effects require further investigation due to small sample sizes and potential publication bias. Practically, these findings provide preliminary evidence that consuming 3–6 mg/kg of caffeine approximately 1 h before plyometric CA may maximize performance enhancement.

## 1 Introduction

Post-activation performance enhancement (PAPE) refers to the acute improvement in sports performance (e.g., sprinting, jumping, or throwing) elicited by a preceding bout of appropriately intense conditioning activity (CA) (1–4). Evidence suggests that PAPE typically occurs within 2.5–11 min following the CA, with peak effects observed around 5.5 min post-CA (2). It should be noted, however, that the timing of peak effects at ~5.5 min can be influenced by multiple factors, including the type of CA performed, participant characteristics, and the comprehensiveness of the warm-up (1–3, 5–7). Studies have reported that PAPE can improve performance by 2–10% (1–3, 5–7). Given its effectiveness, PAPE has been widely incorporated into warm-up protocols and complex training programs to optimize acute sports performance and facilitate long-term training adaptations (2–4).

The PAPE results from a balance between performance enhancement and fatigue (1, 2, 8), suggesting that the exercise performed to induce PAPE has to be intense enough to elicit neuromuscular excitation, while allowing sufficient recovery time to avoid fatigue dampening the potentiation effect (2, 7, 9). Given that most current CA protocols use maximal or near-maximal loads, they may rapidly deplete ATP in the phosphagen energy system and induce considerable fatigue (1, 2, 4, 10). This raises the practical challenge of how to attenuate fatigue while preserving or even amplifying the potentiation response. One potential strategy is the use of nutritional or ergogenic aids, which have therefore received growing attention (11–14). Common supplements (e.g., caffeine, creatine, and carbohydrate mouth rinses) may act through different mechanisms: caffeine and carbohydrate mouth rinses have been shown to enhance central nervous system excitability (11), whereas creatine primarily increases intramuscular phosphocreatine availability and supports energy metabolism (12, 14). Collectively, these effects may contribute to a more favorable balance between performance enhancement and fatigue, thereby maximizing the performance benefits of PAPE protocols (1, 2).

Different types of supplements may influence the balance between performance enhancement and fatigue through distinct physiological mechanisms (15). For example, caffeine primarily acts on the central nervous system by antagonizing adenosine receptors, thereby enhancing neurotransmitter release, increasing motor unit firing rates, and reducing the perception of pain (16). Creatine supplementation increases intramuscular phosphocreatine stores, facilitating faster ATP resynthesis during recovery and between training bouts (12). In contrast, carbohydrate mouth rinses may exert their effects by stimulating oral taste receptors and activating central neural pathways associated with motor output (14).

Previous studies comparing PAPE with supplements to PAPE with placebo (PLA) have reported mixed findings (17–26). For instance, Heydari et al. (17) found that caffeine improved vertical jump height and minimum power output during repeated sprints, but had no significant effects on standing long jump or total sprint time, peak power, and average power. Other studies showed no clear benefit of creatine or carbohydrate supplementation on maximal strength or repeated sprint performance (22, 23, 25, 26). These inconsistencies may stem not only from methodological limitations such as small sample sizes, differing designs, and varied outcome measures, but also from genuine heterogeneity in treatment effects across participant characteristics, supplement types, CA types, and performance outcomes (2).

**Figure 1:**
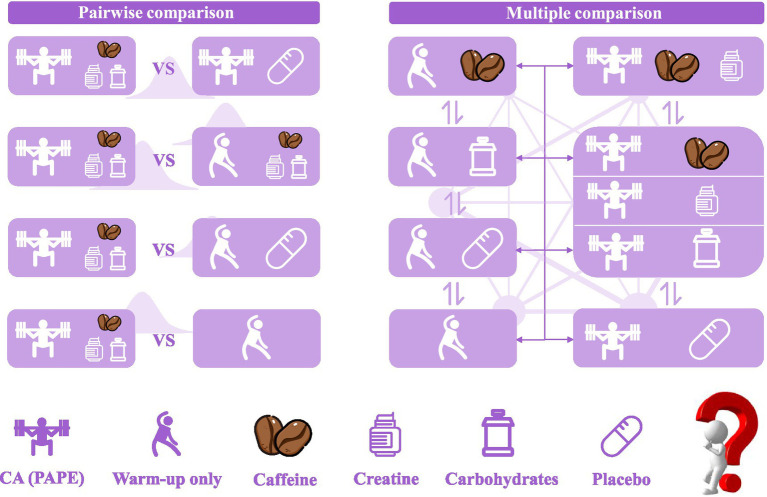
Pairwise comparison and multiple comparison diagram. CA, conditioning activity; the left background indicates the use of Bayesian methods, while the right background and bidirectional arrows indicate that a network multiple comparison was conducted.

In addition to PAPE + supplement vs. PLA comparisons, other intervention strategies, including PAPE with two supplements, warm-up (i.e., general warm-up for non-PAPE) with supplements, warm-up with PLA, and warm-up alone, form a complex network of multiple comparisons (Figure 1). Many studies also report multiple performance outcomes, limiting the statistical power of traditional meta-analyses focused on single outcomes. Therefore, this study adopts a multilevel Bayesian meta-analytic approach, incorporating both pairwise and multiple comparisons (Figure 1), to address the nested data structure. (i) evaluate the effects of PAPE combined with supplements on sports performance across various intervention strategies, and specifically assess its fatigue resistance compared to PAPE combined with PLA; and (ii) synthesize direct and indirect evidence on sports performance outcomes using network meta-analysis to improve the precision of effect estimates. We hypothesized that combining supplements with PAPE would provide superior performance benefits compared to PAPE + PLA, with caffeine showing the greatest enhancement due to its central nervous system effects.

## 2 Methods

The systematic review followed the PRISMA (Preferred Reporting Items for Systematic Reviews and Meta-Analyses) 2020 guidelines (27). Study titles were preregistered in the Open Science Framework^1^ before data analysis.

### 2.1 Eligibility criteria

The inclusion criteria were established based on the PICOS framework. Participants (P): Studies involving at least recreationally active individuals (28), regardless of age or sex, were included; Intervention (I): The intervention group was required to include a combination of a CA, typically designed to elicit PAPE, and a supplement. Examples include PAPE combined with caffeine, creatine, or carbohydrate mouth rinse; Comparison (C): The control groups included combinations such as PAPE + PLA, warm-up + caffeine, warm-up + PLA, or warm-up alone; Outcomes (O): Eligible outcome measures were those reflecting the magnitude of the PAPE effect, including tests of sports performance (e.g., jump performance, sprint performance, Wingate performance, repeated sprint ability, sport-specific performance tests, and medicine ball throw), as well as fatigue resistance outcomes, such as the fatigue index calculated as the relative decline in performance across repeated sets compared to the best set (29); Study design (S): Only randomized controlled trials or randomized crossover studies published in peer-reviewed English-language journals were included. No additional eligibility criteria were applied for the network meta-analysis; it was conducted based on the studies that had already been included according to the above paired comparison criteria. The included studies followed the classical acute PAPE experimental framework, which typically does not impose restrictions on study duration or sample size (2).

### 2.2 Search strategy

Searches were conducted in three English databases (Web of Science, SPORTDiscus, and PubMed). Additional studies were identified by reviewing published caffeine, creatine, and carbohydrate mouth rinses, utilizing Google Scholar, and examining citations of included studies. The search was independently conducted by two reviewers (Y. L. and K. X.), covering the period from database inception to June 21, 2025. Boolean phrases and keywords used are detailed in Appendix B.

### 2.3 Study selection

One reviewer (Y. L.) initially identified relevant studies and determined which titles and abstracts to include. During the eligibility phase, two reviewers (Y. L. and B. Q.) independently assessed the full texts based on the inclusion and exclusion criteria. In cases of uncertainty, a third reviewer (K. X.) was consulted for the final decision. All processes were conducted using EndNote reference manager (version 20; Clarivate Analytics, Philadelphia, PA, United States).

### 2.4 Data extraction

Data extraction was independently conducted by two reviewers (Y. L. and H. K.), with the extracted data reviewed and validated by two additional reviewers (K. X. and B. Q.). Data from the included studies were organized in Excel^®^ (Microsoft Corporation, Redmond, WA, United States) under the following categories: (i) basic information: authors, publication date, sample size, sex, age, height, body mass, training experience, and training level; (ii) intervention information: CA type, CA volume (i.e., sets, repetitions, rest time between sets), warm-up, supplement dosage; (iii) outcomes: jump performance (i.e., countermovement jump and standing long jump height and peak power), linear sprint time, repeated sprint ability (i.e., total sprint time, mean sprint time, peak sprint time, mean power, peak power, mean velocity, peak velocity, fatigue index), 30-s Wingate performance (i.e., peak power, mean power, total power, and fatigue index), medicine ball throw distance, strength, and specific performance (i.e., taekwondo-specific agility test, 10s frequency speed of kick test, multiple frequency speed of kick test, and fatigue index); (iv) other information: recovery time, experimental design (randomized controlled and crossover design), feeding status, and comparison of types (i.e., whether outcomes were measured only once after the CA, or both before and after). For studies in which data were presented in graphical form, numerical values were extracted using WebPlotDigitizer (version 4.5; https://www.colliseum.net/WebPlot/).

For pairwise comparisons, Participants’ training levels were classified as recreationally active (Tier 1), trained (Tier 2), and highly trained (≥ Tier 3) (28). For the network meta-analysis involving multi-arm comparisons, intervention categories were defined as follows: PAPE + caffeine (PAPE + CAF), PAPE + creatine (PAPE + CRE), PAPE + carbohydrate mouth rinse (PAPE + CHO), PAPE + caffeine + creatine (PAPE + CAF + CRE), PAPE + placebo (PAPE + PLA), warm-up + caffeine (WU + CAF), warm-up + carbohydrate mouth rinse (WU + CHO), warm-up + placebo (WU + PLA), and warm-up alone (WU). Data coding was independently performed by two reviewers (Y. L. and B. Q.). Any discrepancies were resolved through discussion with a third reviewer (K. X.) until consensus was reached.

### 2.5 Risk of bias evaluation and certainty assessment

The risk of bias was evaluated using the Cochrane Risk of Bias 2.0 (RoB-2) tool (30). Two reviewers independently assessed five key domains: the randomization process, deviations from intended interventions, missing outcome data, outcome measurement, and selection of the reported results. To assess the certainty of the evidence, the Grading of Recommendations Assessment, Development and Evaluation (GRADE) framework was employed (31, 32). The starting level of certainty was considered high and could be downgraded based on the following factors: limited total sample size (≤400 participants), considerable between-study heterogeneity (*I^2^* > 50%), ambiguity in the direction of the pooled effect, and indications of publication bias. All evaluations were conducted independently by two reviewers (Y. L. and K. X.), with any discrepancies resolved through discussion to achieve consensus.

### 2.6 Statistical analysis

In a preliminary screening conducted prior to the formal systematic search, we found that only a limited number of studies met the predefined inclusion and exclusion criteria. Therefore, a multilevel Bayesian meta-analytic approach was adopted (33). The Bayesian framework was chosen because it avoids the dichotomous interpretation inherent in frequentist hypothesis testing (e.g., declaring significance based on *p*-values) and instead focuses on estimating the posterior distribution of the effect size (ES), allowing for more informative questions, such as the probability that the effect size exceeds a meaningful threshold (33–35). Moreover, Bayesian models are particularly well-suited for small sample sizes (33). Given that most included studies reported only one post-CA measurement, we extracted and analyzed post-CA test outcomes for all comparisons. In response to the reviewer’s comment, we additionally conducted a sensitivity analysis by combining results based on change scores and post-CA measurements, assuming pre-post correlations of 0.6, 0.7, 0.8, and 0.9. ES were computed using the “escalc” function from the *metafor* package, which includes small-sample bias correction by default (36).

For pairwise comparisons, we employed weakly informative priors as recommended by Williams et al. (37). Specifically, the prior distribution for the overall effect (*μ*) was set as a normal distribution with mean 0 and variance 1 (μ ∼ N(0, 1)), and the heterogeneity parameter (*τ*) was given a Half-Cauchy prior with location 0 and scale 0.5 (τ ∼ HC(0, 0.5)) (34, 37). Since many studies reported multiple outcome measures and some employed crossover designs, a hierarchical data structure was implemented with observations nested within groups, and groups nested within studies (38). To account for the correlation between repeated measurements on the same participants, a variance–covariance matrix was constructed assuming a correlation coefficient of 0.8. Posterior inference was performed using Hamiltonian Markov Chain Monte Carlo sampling, and results were reported as posterior means along with 95% credible intervals (Crl). The primary model evaluated the following four comparisons:

- PAPE + supplement vs. PAPE + PLA;
- PAPE + supplement vs. warm-up + supplement;
- PAPE + supplement vs. warm-up + PLA;
- PAPE + supplement vs. warm-up.

For comparison (i), the main analyses were conducted based on performance outcomes and fatigue resistance outcomes. In addition, to explore potential sources of heterogeneity and moderating factors, subgroup analyses were performed on the performance outcomes from comparison (i) according to CA type, supplement type, participants’ training level, and performance outcome type. Model fit and predictive performance were evaluated using multiple Bayesian diagnostic tools. Posterior predictive checks were performed using the “pp_check” function to visually assess model adequacy (34). Leave-one-out cross-validation was conducted via the “loo” function to estimate the expected log predictive density for each observation, and corresponding Pareto k values were used to identify potentially influential data points (38). After excluding effect sizes with Pareto k values greater than 0.7, the model was refitted to assess the robustness of the results. Heterogeneity (*I^2^*) was calculated at three hierarchical levels (i.e., observations, groups, and studies) based on the posterior distributions of the corresponding variance parameters (34, 38). ES values were categorized as *small* (<0.2), *moderate* (0.2–0.49), *large* (0.5–0.8) or *very large* (>0.8), and *I^2^* values of 25, 50, and 75% were interpreted as indicating low, moderate, and high heterogeneity, respectively.

Subsequently, to compare all intervention strategies (e.g., PAPE+CAF, PAPE+CRE, PAPE+CHO, PAPE+CAF + CRE, PAPE+PLA, WU + CAF, WU + CHO, WU + PLA, WU), we conducted a multilevel Bayesian network meta-analysis using contrast-based data. This approach was necessary due to the predominance of three-arm or multi-arm trials in the included studies (17–23), which are not easily handled by pairwise models. A random-effects model was fitted using the “nma” function from the *multinma* package, with default prior settings (39). To account for dependencies between multiple effect sizes from the same group of participants (e.g., across different outcomes or time points), we applied a multilevel structure by aggregating effect sizes using the “aggregate” function from the *metafor* package, assuming a within-cluster correlation of 0.8 (40). Both the variance–covariance matrix and the within-cluster correlation were determined based on the known structure of the original data (unpublished) and previous meta-analyses (2). Furthermore, we conducted posterior relative effects analysis using “WU” and “PAPE+PLA” as reference comparators and ranked the intervention groups based on the Surface Under the Cumulative Ranking curve (SUCRA), which quantifies the probability of each intervention being among the most effective (34, 35).

To quantify the probability of a positive overall effect, we used the empirical cumulative distribution function to calculate the posterior probability that the pooled ES exceeded zero. Risk of publication bias was assessed using funnel plots and Egger’s regression test. All analyses were conducted using R (version 4.3.0; R Core Team, Vienna, Austria). Pairwise Bayesian models were implemented with the brms package (41), network meta-analysis was performed with the *multinma* package (39), and graphical outputs were generated using *ggplot2* (42). All analysis code can be found at https://osf.io/a7d5n/.

## 3 Results

### 3.1 Search result and study characteristics

**Figure 2:**
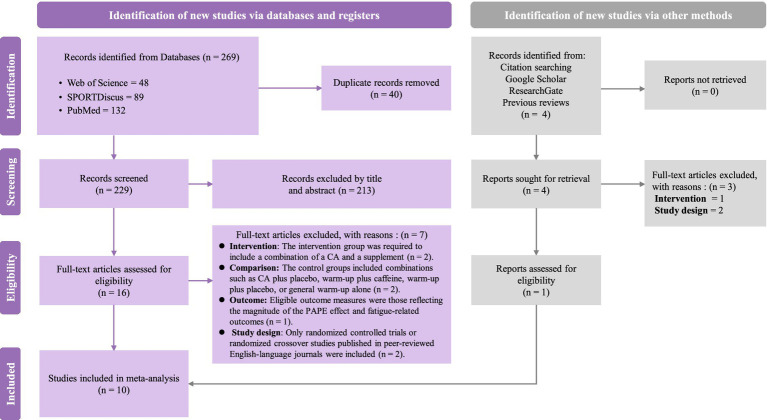
PRISMA flow diagram of study selection.

A total of 269 articles were retrieved from three databases. After screening, nine articles met the inclusion and exclusion criteria, and one additional article was included through other sources, resulting in a total of 10 studies (Figure 2). Among these, three studies employed image extraction tools.

Study characteristics are presented in Tables 1, 2. A total of 198 participants were included: 24 females, 160 males, and 14 with unspecified sex. Participant age ranged from 16.4 ± 1.1 to 34.6 ± 7.0 years, body mass from 59.2 ± 10.0 to 80.0 ± 12.0 kg, and height from 1.68 ± 0.09 to 1.78 ± 0.04 m. Training experience ranged from 0.5 to 13 years. One study involved recreationally active participants, three involved trained individuals, and six involved highly trained individuals. Eight studies employed a randomized crossover design, and two used a randomized controlled design. Five studies conducted both pre-CA and post-CA tests, while the other five conducted post-CA testing only.

**Table 1:** Participant characteristics.

Study	*N*	Age	Body mass	Height	Level	Training experience
Heydari et al. (17)	20 M	34.6 ± 7.0	68.8 ± 5.6	1.76 ± 0.05	RA	5.8 ± 2.3
Zhang et al. (18)	30 M	20.0 ± 1.0	80.0 ± 12.0	1.78 ± 0.04	HT	5 ± 1
Huerta Ojeda et al. (19)	7 M	17.4 ± 1.2	66.9 ± 4.2	1.71 ± 0.05	HT	>2 y
	7 M	17.1 ± 0.8	69.0 ± 6.4	1.73 ± 0.08	HT	>2 y
	7 M	17.4 ± 0.9	68.0 ± 5.2	1.72 ± 0.06	HT	>2 y
	7 M	16.7 ± 0.7	70.2 ± 8.4	1.73 ± 0.08	HT	>2 y
Ouergui et al. (20)	20 (10 M, 10F)	17.5 ± 0.7	59.2 ± 10.0	1.68 ± 0.09	HT	>6 y
Filip-Stachnik et al. (21)	14F	26.0 ± 3.0	62.6 ± 5.6	1.71 ± 0.05	HT	13 ± 3
de Oliveira et al. (22)	14 U	20.9 ± 1.5	77.1 ± 6.9	1.77 ± 0.83	TR	>1 y
Oliveira et al. (23)	20 M	18.9 ± 0.9	71.8 ± 5.2	1.78 ± 0.06	HT	>2 y
Guerra et al. (24)	12 M	23.0 ± 5.0	79.5 ± 5.1	NA	HT	NA
Wang et al. (25)	9 M	16.4 ± 1.1	65.3 ± 7.1	1.72 ± 0.04	TR	>0.5 y
	8 M	16.8 ± 0.7	65.3 ± 4.7	1.69 ± 0.04	TR	>0.5 y
Wang et al. (26)	15 M	19.9 ± 1.9	70.0 ± 6.7	1.72 ± 0.05	TR	>0.5 y
	15 M	19.5 ± 1.1	70.2 ± 11.1	1.76 ± 0.09	TR	>0.5 y

N, sample size; M, male; F, female; RA, recreationally active; HT, highly trained; TR, trained; y, year; U, unclear; NA, Not applicable.

**Table 2:** The characteristics of the studies included.

Study	Design	Conditions	PAPE	WU	Supplementation; form	Feeding status	Recovery time	Outcome
Heydari et al. (17)	RCD Post-T	(1) WU + PLA (2) PAPE + PLA (3) WU + CAF (4) PAPE + CAF	2 × 8 squat jumps 2 × 8 scissor jumps 2 × 8 double-leg bounds (rest between set: 60s; between exercise: 60s)	10 min jogging 5 min dynamic stretch	Before exercise 1 h CAF: 6 mg/kg PLA: 6 mg/kg starch; Capsules	Test day: Same breakfast (350–400 kcal) The day before the test: calorie: 35 kcal/kg body mass carbs: 60–65%, protein: 15–20%, Fat: 20%	5, 10, 15, 20 min	CMJ SLJ sprint RSA
Zhang et al. (18)	RCD Post-T	(1) PAPE + CAF (2) PAPE + PLA (3) WU	8.5% body weight cycling 10s	60w cycling 5 min	before exercise 1 h CAF: 3 mg/kg PLA: 3 mg/kg maltodextrin; Capsules	Test day: last meal: ≥2 h before test The day before the test: no standardized dietary control; but abstain from caffeine, alcohol, and additional supplements in the previous 24 h	2 or 10 min	30 s Wingate test
Huerta Ojeda et al. (19)	RCT Pre-Post-T	(1) PAPE + CAF + CRE (2) PAPE + PLA + CRE (3) PAPE + CAF + PLA (4) PAPE + PLA + PLA	1 × 4 × 30%1RM BS 1 × 4 × 60%1RM BS	1 × 10 plantar flexion and dorsiflexion of the ankles; 1 × 10 flexion and extension of the knees; 1 × 10 flexion and extension of the hips; 1 × 10 flexion, extension, adduction, and abduction of the shoulders; 6 min jogging 2 × 10 s of leg, thigh, and hip muscle stretches	CRE: 0.3 g/day for 14 days CAF: 0.3 mg/kg ingested 1 h before exercise PLA: 0.3 g/day for 14 days maltodextrin; CRE: powder CAF: liquid solution	Test day: last meal: ≥2 h before test The day before the test: no standardized dietary control, but must abstain from caffeine, alcohol, carbonated drinks, protein shakes, and metabolic activators; maintain the Everton Club’s regular diet guidelines	1 min	RSA
Ouergui et al. (20).	RCD Post-T	(1) WU + PLA (2) WU + CAF (3) PAPE + PLA (4) PAPE + CAF (5) WU	3 × 10 vertical jumps (rest between set: unclear)	10 min jogging	before exercise 1 h CAF: 3 mg/kg· PLA: 3 mg/kg·unclear; Liquid solution;	Test day: no fasting requirement mentioned The day before the test: no standardized dietary control, but prohibited from caffeine, alcohol, high-intensity exercise, and supplements in the prior 48 h	10 min	TSAT FSKT-10s FSKT-mult
Filip-Stachnik et al. (21).	RCD Pre-Post-T	(1) PAPE + CAF (2) PAPE + PLA (3) WU	1 × 10VL 80%1RM BS	5 min cycling 10 body-weight squats 10 trunk rotations 10 side-bends 10 internal, external and lateral arm swings 5 split stance squats (2 circuits)	Before exercise 1 h CAF: 6 mg/kg PLA: 6 mg/kg all-purpose flour Capsules	Test day: last meal: ≥2 h before test The day before the test: no standardized dietary control, but must abstain from caffeine, alcohol, and additional supplements in the prior 24 h	2, 4, 6, 8, 10 min	CMJ
de Oliveira et al. (22).	RCD Post-T	(1) WU + PLA (2) WU + CHO (10s) (3) WU + CHO (40s) (4) PAPE + PLA (5) PAPE + CHO (10s) (6) PAPE + CHO (40s)	1 × 3 90%1RM BS	5 min jogging	Before RSA test 1 min CHO: rinse the mouth for 10s and 40s 6% (w/v) maltodextrin PLA: sucralose Rinse your mouth for 10 s or 40 s, then spit it out. Do not swallow.	Test day: last meal: ≥2 h before test no alcohol/caffeine during the study period The day before the test: no standardized dietary control, but must abstain from alcohol and caffeine	1 or 8 min	RSA
Oliveira et al. (23)	RCD Post-T	(1) WU + PLA (2) WU + CHO (10s) (3) PAPE + PLA (4) PAPE + CHO (10s)	2 × 5 80%1RM BS (rest between set: 2 min)	5 min jogging	Before RSA test 1 min CHO: rinse the mouth for 10s 6% (w/v) maltodextrin PLA: sucralose; Rinse your mouth for 10 s or 40 s, then spit it out. Do not swallow.	Test day: last meal: ≥2 h before test no alcohol/caffeine during the study period The day before the test: no standardized dietary control, but must abstain from alcohol and caffeine	1 or 8 min	RSA
Guerra et al. (24)	RCD Pre-Post-T	(1) PAPE + CAF (2) PAPE + PLA	2 × 15 ankle hops; 3 × 5 hurdle hops; 1 × 3 20-m sprints with sled towing	1 × 10 forward lunges each side; 3 min dynamic stretching of relevant lower limb musculature; 5 submaximal CMJs	before exercise 1 h CAF: 5 mg/kg PLA: sweetened water; Liquid solution	Test day: breakfast provided by the club (unified dietary prescription); The day before the test: follow the club’s unified meal arrangements (food provided by club staff); prohibited from caffeine, alcohol, and additional supplements (within 24 h before test)	1, 3, 5 min	CMJ
Wang et al. (25).	RCD Pre-Post-T	(1) PAPE + CRE (2) PAPE + PLA	1 × 3 RM bench press	5 min jogging dynamic stretching 2 sets light resistance bench press	CRE: 5 g/day for 6 days; PLA: 5 g/day for 6 days carboxymethyl cellulose; Powder	Test day: no specified fasting/last meal interval The day before the test: maintain normal dietary patterns	Optimal individual time	1RM Medicine ball throw
Wang et al. (26)	RCT Pre-Post-T	(1) PAPE + CRE (2) PAPE + PLA	1 × 5 RM BS	5 min jogging dynamic stretching 2 sets light resistance bench press	CRE: 5 g/day for 6 days; PLA: 5 g/day for 6 days carboxymethyl cellulose Powder	Test day: no specified fasting/last meal interval The day before the test: maintain normal dietary patterns	Optimal individual time	1RM CMJ

WU, warm-up; PLA, placebo; CAF, caffeine; CRE, Creatine; PAPE, post-activation performance enhancement; CMJ, countermovement jump; SLJ, standing long jump; RSA, repeated sprint ability; BM, body mass; RM, repetition maximum; BS, back squat; TSAT, taekwondo-specific agility test; FSKT-10s, 10 s frequency speed of kick test; FSKT-mult, multiple frequency speed of kick test; CHO, carbohydrate Mouth Rinse; RCD, randomized crossover design; RCT, randomized controlled trial; Post-T, measured only once after the conditioning activity; Pre-Post-T, A measurement was taken before and after the conditional activity.

Intervention conditions included the following (number of studies, k): PAPE + CAF (*k* = 6), PAPE + CRE (*k* = 3), PAPE + CHO (*k* = 2), WU + CAF (*k* = 2), PAPE + CAF + CRE (*k* = 1), PAPE + PLA (*k* = 10), WU + PLA (*k* = 3), WU + CHO (*k* = 2), and WU only (*k* = 2). PAPE protocols consisted of bodyweight exercises (*k* = 3), resistance training (*k* = 6), and loaded cycling (*k* = 1). Regarding warm-up protocols, four studies used jogging only, one study used jogging and stretching, and five studies included jogging, stretching, bodyweight exercises, and sport-specific warm-up.

Caffeine was administered 1 h before exercise at doses of 3 mg/kg (*k* = 2), 5 mg/kg (*k* = 1), and 6 mg/kg (*k* = 2); one study (*k* = 1) used 0.3 mg/kg. CRE supplementation was administered as 0.3 g/kg per day for 14 days (*k* = 1) or 5 g per day for 6 days (*k* = 2). Carbohydrate mouth rinse was administered via mouth rinsing with 6% (w/v) maltodextrin for 10 s (*k* = 1) or 40 s (*k* = 2). Recovery durations varied: five studies used a fixed single time point, three used multiple time points, and two selected the optimal time point. Outcome measures included jump performance (*k* = 4), linear sprint performance (*k* = 1), repeated sprint ability (*k* = 4), 30-s Wingate performance (*k* = 1), medicine ball throw distance (*k* = 1), maximal strength (*k* = 2), and sport-specific performance (*k* = 1).

### 3.2 Risk of bias assessment

**Figure 3:**
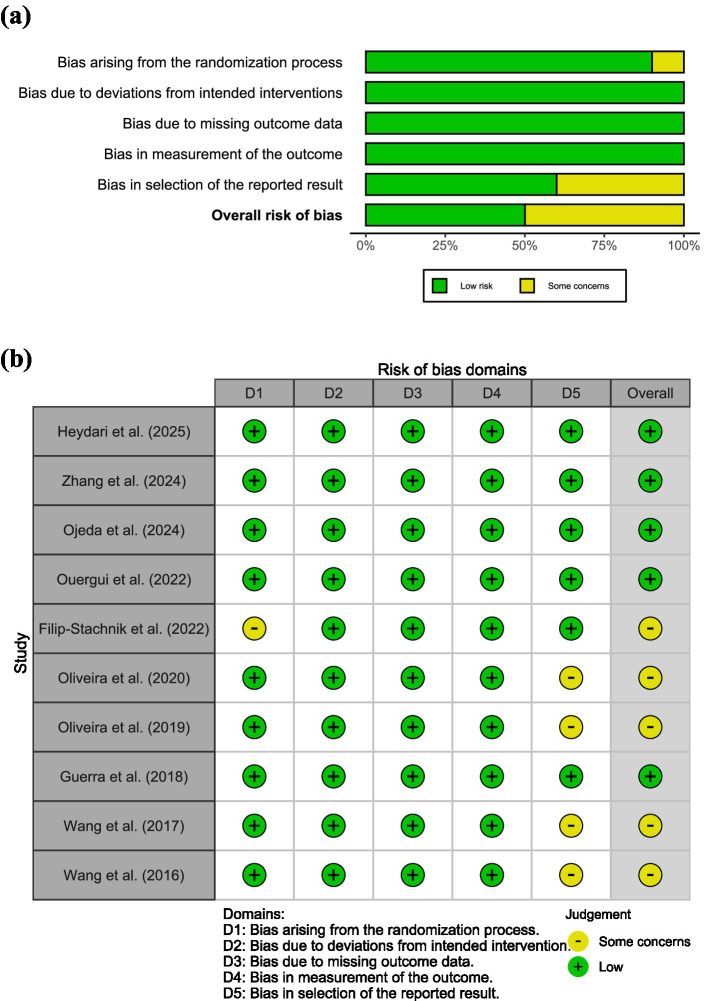
Risk of bias assessment. **(a)** Risk of bias summary plot. **(b)** Risk of bias traffic light plot.

Regarding randomization, nine studies employed both randomization and double-blinding, and were therefore judged to have a “low” risk of bias. One study used randomization alone without blinding, resulting in a judgment of “some concerns.” In addition, both de Oliveira et al. (22), Oliveira et al. (23), and Wang et al. (25, 26) published two similar studies, raising the possibility of selective reporting and thus were also rated as having “some concerns” in that domain. All other domains were rated as “low” risk. Overall, 50% of the studies were assessed as having a “low” risk of bias, while the remaining 50% were judged to have “some concerns” (Figure 3).

### 3.3 Pairwise comparison results

**Figure 4:**
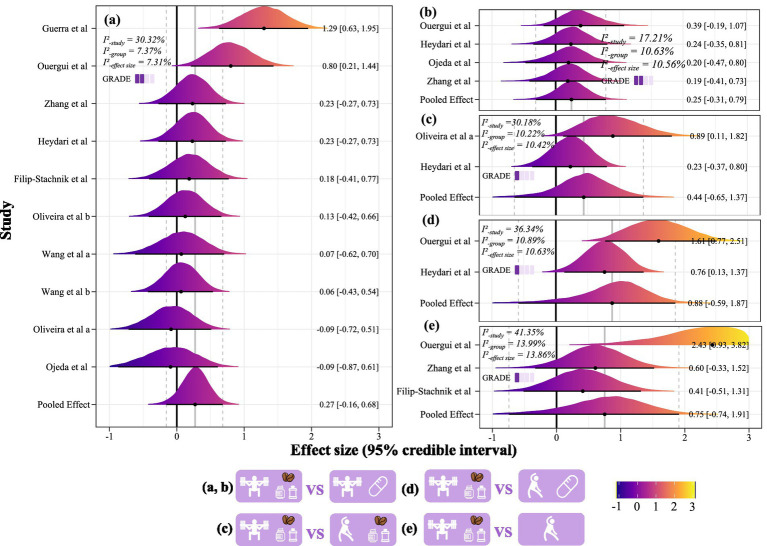
The effects of PAPE + supplement compared to PAPE + placebo on sports performance **(a)** and fatigue index **(b)**; The effects of PAPE + supplement compared to warm-up + supplement **(c)**, warm-up + PLA **(d)**, and warm-up alone **(e)** on sports performance.

Compared to PAPE combined with PLA, PAPE combined with supplements (i.e., caffeine, creatine, and carbohydrate mouth rinse) showed a 90.83% probability of improving performance outcomes (ES = 0.27, 95% CrI: −0.16 to 0.68; *I^2^*-study = 30.32%, *I^2^*-group = 7.37%, *I^2^*-effect size = 7.31%; GRADE = low; Figure 4a) and an 83.65% probability of enhancing fatigue resistance (ES = 0.25, 95% CrI: −0.31 to 0.79; *I^2^*-study = 17.21%, *I^2^*-group = 10.63%, *I^2^*-effect size = 10.56%; GRADE = very low; Figure 4b). No significant risk of publication bias was detected (*p* = 0.86 and 0.58, Supplementary Figure S1). Sensitivity analyses (Supplementary Figures S5–S7) showed that when assuming pre-post correlations of *r* = 0.9 (ES = 0.36; 95% CrI: 0.01 to 0.71), *r* = 0.8 (ES = 0.31; 95% CrI: 0.01 to 0.60), *r* = 0.7 (ES = 0.28; 95% CrI: 0.00 to 0.55), and *r* = 0.6 (ES = 0.27; 95% CrI: −0.00 to 0.53), the findings for performance outcomes were consistent. Results for fatigue resistance outcomes were unchanged across all assumptions (all ES = 0.25). Moreover, after excluding ESs with Pareto k values greater than 0.7, performance outcomes remained similar (ES = 0.35; 95% CrI: −0.14 to 0.82), whereas the ES for fatigue resistance was substantially attenuated, being supported by only a single study (ES = −0.00; 95% CrI: −1.27 to 1.23).

The subgroup analysis indicated that CA type was the primary source of heterogeneity (I^2^-study = 20.17%). Plyometric CA (i.e., jump and sprint) combined with supplementation (all were caffeine) was more effective than plyometric CA + PLA (ES = 0.97, >0 probability = 99.79%). Moreover, this approach was also more effective than resistance training combined with supplementation (i.e., caffeine, creatine, and carbohydrate mouth rinse; ES = 1.06, >0 probability = 99.65%). In contrast, the results were similar across supplement types, training levels, and performance outcomes (Table 3).

**Table 3:** Subgroup analysis.

Subgroup variables	ES 95% Crl	>0 probability	*I^2^*-study	*I^2^*-group	*I^2^*-effect size
Type of supplement					
Caffeine	0.33 [−0.20, 0.81]	90.69%	31.89%	7.18%	6.98%
Carbohydrate mouth rinse	−0.10 [−1.12, 0.90]	40.94%			
Creatine	0.49 [−0.04, 1.00]	96.65%			
Type of conditioning activity					
Plyometric jump and sprint	0.97 [0.42, 1.54]	99.79%	**20.17%**	**8.45%**	**8.38%**
Cycling	0.22 [−0.72, 1.17]	69.50%			
Resistance training	−0.09 [−0.52, 0.33]	32.22%			
Training level					
Highly trained	0.22 [−0.48, 0.90]	75.39%	34.18%	6.97%	6.87%
Trained	0.36 [−0.47, 1.14]	82.90%			
Recreationally active	0.20 [−1.35, 1.68]	61.19%			
Performance outcomes					
Throw	0.15 [−1.08, 1.43]	59.48%	30.18%	7.72%	7.70%
Vertical jump	0.31 [−0.30, 0.84]	85.19%			
Long jump	0.01 [−0.73, 0.76]	51.30%			
Repeated sprint ability	0.10 [−0.44, 0.63]	65.14%			
Taekwondo Specialized Test	1.07 [−0.36, 2.44]	94.20%			
Linear sprint	−0.13 [−0.88, 0.61]	35.69%			
Strength (1RM)	0.38 [−0.64, 1.43]	76.97%			
30s Wingate test	0.20 [−1.18, 1.55]	63.66%			

ES, effect size; Crl, confidence interval; bold indicates heterogeneous sources.

Compared to warm-up combined with supplements (ES = 0.44, 95% CrI: −0.89 to 1.37; *I^2^*-study = 30.18%, *I^2^*-group = 10.22%, *I^2^*-effect size = 10.42%; GRADE = very low; Figure 4c), PAPE combined with supplements showed an 85.09% probability of improving sports performance. When compared to warm-up combined with PLA (ES = 0.88, 95% CrI: −0.59 to 1.87; *I^2^*-study = 36.34%, *I^2^*-group = 10.89%, *I^2^*-effect size = 10.63%; GRADE = very low; Figure 4d), the probability of improvement was 92.29%. Compared to warm-up only (ES = 0.75, 95% CrI: −0.74 to 1.91; *I^2^*-study = 41.35%, *I^2^*-group = 13.99%, *I^2^*-effect size = 13.86%; GRADE = very low; Figure 4e), the probability was 88.10%. All three comparisons showed a significant risk of publication bias (*p* < 0.01, Supplementary Figures S1, S4). Sensitivity analyses excluding influential data points showed no meaningful changes in the posterior distributions or conclusions, supporting the robustness of the original model (Supplementary Figures S8, S9).

### 3.4 Multiple comparison results

**Figure 5:**
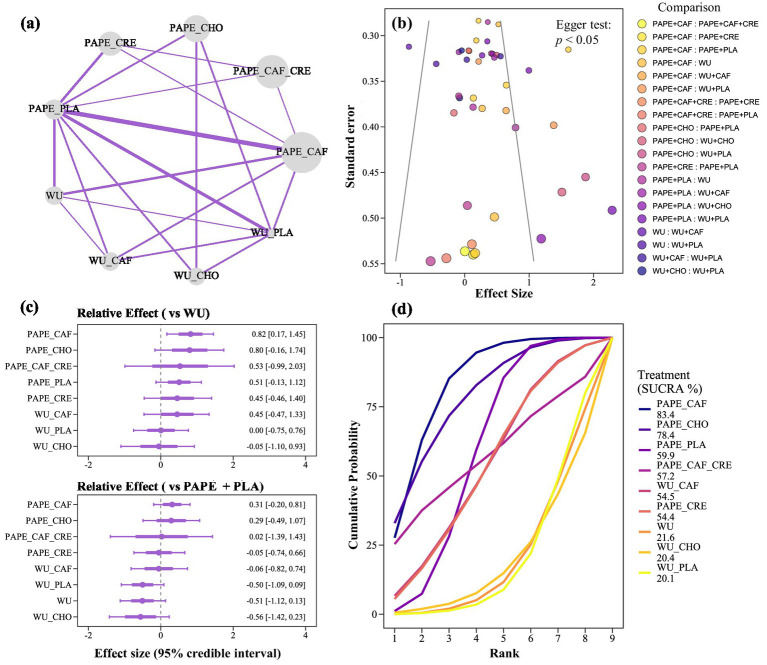
**(a)** Network diagram of multiple comparisons. **(b)** Risk of publication bias in multiple comparisons. **(c)** Results of multiple comparisons based on the WU group and PAPE + PLA. **(d)** SUCRA ranking. SUCRA, surface under the cumulative ranking curve. In panel **(a)**, the size of each node corresponds to the sample size of the comparison.

The Bayesian model fit was acceptable, with a residual deviance of 22.6 (based on 23 data points), an effective number of parameters of 19.7, and a DIC value of 42.3 (Supplementary Figures S10–S13). Figure 5a presents the network plot. Based on the combined results of direct and indirect comparisons, the probability that PAPE + CAF improves performance compared to WU and PAPE + PLA was 99.17% (ES = 0.82, 95% CrI: 0.17 to 1.45) and 89.75% (ES = 0.31, 95% CrI: −0.20 to 0.81; Figure 5c), respectively. Similarly, PAPE + CHO showed a 95.12% probability of improving performance compared to WU (ES = 0.80, 95% CrI: −0.16 to 1.74; Figure 5c) and a 77.30% probability compared to PAPE + PLA (ES = 0.29, 95% CrI: −0.49 to 1.07; Figure 5c). According to the SUCRA rankings, the top two interventions were PAPE + CAF (83.40%; Figure 5d) and PAPE + CHO (78.40%; Figure 5d). Overall, there was a significant risk of publication bias (*p* < 0.01; Figure 5b).

## 4 Discussion

To the best of our knowledge, this is the first meta-analysis to examine the effects of combining PAPE with supplements on sports performance. Based on pairwise comparisons, the probability that PAPE combined with supplements results in a positive effect (ES > 0) on sports performance was 90.83% compared to PAPE + PLA, 85.09% compared to warm-up + supplement, 92.29% compared to warm-up + PLA, and 88.10% compared to warm-up alone. In addition, the probability that PAPE combined with supplements leads to improved fatigue resistance compared to PAPE + PLA was 83.65%. Furthermore, we identified the CA method as a major source of heterogeneity, with plyometric CA (i.e., jump and sprint) combined with caffeine showing the greatest improvement in performance outcomes (e.g., jump, sprint, repeated sprint, specific performance). Multiple comparisons further indicated that PAPE + CAF (SUCRA = 83.40%) and PAPE + CHO (SUCRA = 78.40%) were the most effective strategies for enhancing sports performance, with PAPE + CAF showing a 99.17% probability of outperforming (ES > 0) warm-up alone. Overall, these findings provide preliminary evidence that plyometric CA (PAPE) performed after ingesting 3–6 mg/kg of caffeine 1 h before exercise may be the most effective strategy for enhancing athletic performance, which is consistent with our original hypothesis.

### 4.1 Pairwise comparison

PAPE combined with supplements (i.e., caffeine, creatine, and carbohydrate mouth rinses) demonstrated a 90.83% probability of improving sports performance compared to PAPE + PLA. However, the 95% CrI crossed zero, and heterogeneity was moderate (*I^2^*-study = 30.32%), indicating limited confidence in the superiority of the combination. Furthermore, sensitivity analyses indicated that when combining change scores with post-CA measurements, assuming pre-post correlations of *r* = 0.7–0.9, the 95% CrIs did not cross zero. However, for *r* = 0.6 and post-only comparisons with effect sizes having Pareto *k* > 0.7 excluded, the 95% CrIs crossed zero. These not-robust results further highlight that this field remains in early development. Although Guerra et al. (24) and Ouergui et al. (20) reported relatively large effects in the pooled model, the direction of effect was generally consistent across other models that incorporated the variance–covariance matrix, with heterogeneity substantially reduced. This suggests that over 80% of the included studies reported modest advantages of supplement ingestion compared to placebo. Overall, these findings support the use of a Bayesian pooled approach, but the results should be interpreted with caution due to remaining uncertainty.

A recent study suggested that the effectiveness of PAPE can be influenced by the comprehensiveness of the warm-up; specifically, when the warm-up is both thorough and sport-specific, the added benefit of PAPE may be negligible (2). This suggests a potential upper limit to the performance benefits of warm-up strategies, with diminishing returns (2, 4, 9). While supplementing PAPE appears to further enhance performance, the effect remains modest (ES = 0.27, 95% CrI: −0.16 to 0.68). Among the 10 included studies, Guerra et al. (24) and Ouergui et al. (20) reported the largest effects compared to PAPE + PLA (ES = 1.60 and 1.11, respectively), both using caffeine. Subgroup analyses confirmed that plyometric CA combined with caffeine produced the largest performance gains, suggesting that the high effects observed in Guerra et al. (24) and Ouergui et al. (20) may reflect the unique benefits of this combination rather than being true outliers. Notably, this remains speculative, given the limited number of studies and the instability of the current model estimates, warranting further investigation. Additionally, as other subgroup analyses did not yield meaningful results, they will not be discussed in detail.

We also examined the impact of PAPE + supplements (i.e., caffeine and creatine) on fatigue resistance compared to PAPE + PLA. Although the included studies consistently reported positive trends, the overall probability of improved fatigue resistance was only 83.65%, and after excluding effect sizes with Pareto k values greater than 0.7, the effect was minimal. This uncertainty may be due to (i) the small number of available studies, (ii) the mismatch between the testing time point and the individual’s optimal recovery window post-PAPE (1, 2), or (iii) individual variability in neuromuscular fatigue sensitivity, all of which could obscure the supplements’ fatigue-attenuating effects (2). Mechanistically, caffeine may exert a central nervous system stimulatory effect by antagonizing adenosine receptors (particularly A1 and A2a), thereby promoting alertness, arousal, and reduced pain perception (16, 43). Creatine can support ATP resynthesis by regenerating ATP from ADP via phosphorylation and buffer intracellular pH via H^+^ uptake during the creatine kinase reaction, enhancing cellular homeostasis during high-intensity exercise (12, 44). These theoretical mechanisms may explain the positive trends observed, but given the limited and uncertain evidence, the anti-fatigue effects of PAPE combined with these supplements remain preliminary.

Other pairwise comparisons showed that PAPE + supplements had a probability of improving performance compared to warm-up + supplement, warm-up + PLA, and warm-up alone of 85.09, 92.29, and 88.10%, respectively. These comparisons were limited by small sample sizes, wide CrIs, and moderate heterogeneity, along with a significant risk of publication bias. Based on these limitations, and to address the small number of studies for certain pairwise comparisons, we also conducted a network meta-analysis, which aimed to explore potential trends and generate preliminary insights from the limited available data.

### 4.2 Multiple comparison

In our multilevel Bayesian network meta-analysis, multiple performance outcomes within each intervention group were aggregated to estimate the average effect of each intervention on sports performance. Under this model, PAPE+CAF (SUCRA = 83.40%) and PAPE+CHO (SUCRA = 78.40%) demonstrated the highest probabilities of improving performance. Caffeine may enhance performance by promoting calcium ion mobilization, increasing force production per motor unit, and potentially enhancing peripheral neuromuscular function through upregulation of Na^+^/K^+^ pump activity (11, 16, 19, 43). In contrast, CHO may exert its effects by stimulating taste receptors and activating central neural pathways associated with motor output (14, 22, 23).

However, when comparing against WU (i.e., warm-up alone), only PAPE + CAF exhibited a 95% CrI that excluded zero, with a 99.17% probability of enhancing performance. Interestingly, PAPE + CAF + CRE ranked only fourth in SUCRA, supporting the hypothesis that performance benefits from warm-up strategies may plateau, showing diminishing returns with further supplementation. This suggests that simply increasing supplement dosage or combining multiple supplements does not necessarily yield greater performance gains (19). Instead, identifying the optimal combination of supplement dosage and CA volume may be more effective. Given the considerable inter-individual variability in responses to both CAF and PAPE (16, 45), future studies should explore dose–response interactions to determine personalized strategies for performance enhancement.

### 4.3 Limitations and future research

Given the relatively small overall sample size in this study, we combined all available PAPE-related performance measures and different post-CA time points for analysis. While this approach allowed us to estimate the average effects of each comparison on performance outcomes, it also limited our ability to explore the influence of specific measurement methods and timing. Considerable heterogeneity in CA types, CA volume, supplement types, and dosages among the intervention protocols included in the studies may have influenced the observed effects. Another limitation is the underrepresentation of female participants, who accounted for only 12.12% of the total sample, which greatly restricts the generalizability of our findings to female participants.

Regarding study design, five of the included studies employed post-CA-only measurement designs, while the other five included both pre- and post-CA measurements. According to the Cochrane Handbook, combining change scores with post-only values may lead to biased results (46); therefore, we consistently selected post-CA-only models. However, it should be noted that the pooled results derived from different pre-post correlations were not stable, which substantially limits the clinical significance of PAPE combined with supplements in practice.

In the present study, we attempted to explore potential sources of heterogeneity through subgroup analyses. Although the type of CA emerged as a major contributor to the observed differences, residual heterogeneity remained unexplained. Furthermore, in all models except for PAPE + supplement (i.e., caffeine, creatine, and carbohydrate mouth rinses) versus PAPE + PLA for performance outcomes, a risk of publication bias was detected. To address the issue of limited studies for certain pairwise comparisons, we employed a network meta-analytic approach. Although the overall network model demonstrated good fit and no clear evidence of inconsistency, significant publication bias persisted. These findings suggest that the current reported studies are biased toward positive outcomes, potentially obscuring the true effects of PAPE combined with supplements on performance and fatigue resistance. Therefore, the results of this study should be considered preliminary, and further research is needed to draw more definitive conclusions, including investigations into the potential dose–response relationship between caffeine and PAPE.

Finally, based on the current preliminary findings, to specifically investigate the effects of PAPE, future studies should avoid the concurrent intake of supplements, particularly caffeine, to minimize potential confounding influences.

## 5 Conclusion

Preliminary evidence suggests that combining caffeine with plyometric CA (PAPE) is the most effective strategy for enhancing sports performance. While supplementation with creatine or carbohydrate mouth rinse may offer some benefits, their effects remain inconclusive due to small sample sizes and potential publication bias, and it is still unclear whether PAPE combined with supplements provides greater fatigue resistance compared to PAPE with placebo, warranting further investigation. Notably, simultaneous supplementation of caffeine and creatine on top of PAPE does not appear to produce greater performance improvements, suggesting the presence of marginal returns and an optimal combination strategy when stacking multiple ergogenic aids. Practically, these findings provide preliminary evidence that consuming 3–6 mg/kg of caffeine approximately one hour before plyometric CA may maximize performance enhancement.

## Data Availability

The datasets presented in this study can be found in online repositories. The names of the repository/repositories and accession number(s) can be found in the article/Supplementary material.

## Footnotes

^1^ osf.io/vwzem
