# Supplementary material for: The acute effects of caffeine, creatine, and carbohydrate mouth rinse combined with conditioning activity on subsequent sports performance: a systematic review and Bayesian meta-analysis
Source: Front Nutr. 2025 Nov 6;12:1669004. doi: 10.3389/fnut.2025.1669004 (PMC12631242; doi:10.3389/fnut.2025.1669004)
Supplement: Supplementary file 2 [file Table_2.DOCX]

Supplementary Material

**Appendix A.** PRISMA Checklist.

| **Section and Topic** | **Item #** | **Checklist item** | **Location where item is reported** |
| --- | --- | --- | --- |
| **The acute effects of caffeine, creatine, and carbohydrate mouth rinse combined with conditioning activity on subsequent sports performance: A systematic review and meta-analysis** | | | 1 |
| Title | 1 | Identify the report as a systematic review. | 1 |
| **ABSTRACT** | | |  |
| Abstract | 2 | See the PRISMA 2020 for Abstracts checklist. | 1,2 |
| **INTRODUCTION** | | |  |
| Rationale | 3 | Describe the rationale for the review in the context of existing knowledge. | 2,3 |
| Objectives | 4 | Provide an explicit statement of the objective(s) or question(s) the review addresses. | 3 |
| **METHODS** | | |  |
| Eligibility criteria | 5 | Specify the inclusion and exclusion criteria for the review and how studies were grouped for the syntheses. | 3 |
| Information sources | 6 | Specify all databases, registers, websites, organisations, reference lists and other sources searched or consulted to identify studies. Specify the date when each source was last searched or consulted. | 3 |
| Search strategy | 7 | Present the full search strategies for all databases, registers and websites, including any filters and limits used. | Appendix B |
| Selection process | 8 | Specify the methods used to decide whether a study met the inclusion criteria of the review, including how many reviewers screened each record and each report retrieved, whether they worked independently, and if applicable, details of automation tools used in the process. | 3,4 |
| Data collection process | 9 | Specify the methods used to collect data from reports, including how many reviewers collected data from each report, whether they worked independently, any processes for obtaining or confirming data from study investigators, and if applicable, details of automation tools used in the process. | 4 |
| Data items | 10a | List and define all outcomes for which data were sought. Specify whether all results that were compatible with each outcome domain in each study were sought (e.g. for all measures, time points, analyses), and if not, the methods used to decide which results to collect. | 4 |
|  | 10b | List and define all other variables for which data were sought (e.g. participant and intervention characteristics, funding sources). Describe any assumptions made about any missing or unclear information. | 4 |
| Study risk of bias assessment | 11 | Specify the methods used to assess risk of bias in the included studies, including details of the tool(s) used, how many reviewers assessed each study and whether they worked independently, and if applicable, details of automation tools used in the process. | 4 |
| Effect measures | 12 | Specify for each outcome the effect measure(s) (e.g. risk ratio, mean difference) used in the synthesis or presentation of results. | 5 |
| Synthesis methods | 13a | Describe the processes used to decide which studies were eligible for each synthesis (e.g. tabulating the study intervention characteristics and comparing against the planned groups for each synthesis (item #5)). | 5 |
|  | 13b | Describe any methods required to prepare the data for presentation or synthesis, such as handling of missing summary statistics, or data conversions. | 5 |
|  | 13c | Describe any methods used to tabulate or visually display results of individual studies and syntheses. | 5 |
|  | 13d | Describe any methods used to synthesize results and provide a rationale for the choice(s). If meta-analysis was performed, describe the model(s), method(s) to identify the presence and extent of statistical heterogeneity, and software package(s) used. | 5 |
|  | 13e | Describe any methods used to explore possible causes of heterogeneity among study results (e.g. subgroup analysis, meta-regression). | NA |
|  | 13f | Describe any sensitivity analyses conducted to assess robustness of the synthesized results. | 5 |
| Reporting bias assessment | 14 | Describe any methods used to assess risk of bias due to missing results in a synthesis (arising from reporting biases). | 5,6 |
| Certainty assessment | 15 | Describe any methods used to assess certainty (or confidence) in the body of evidence for an outcome. | 4 |
| **RESULTS** | | |  |
| Study selection | 16a | Describe the results of the search and selection process, from the number of records identified in the search to the number of studies included in the review, ideally using a flow diagram. | 6 |
|  | 16b | Cite studies that might appear to meet the inclusion criteria, but which were excluded, and explain why they were excluded. | 6, 7 |
| Study characteristics | 17 | Cite each included study and present its characteristics. | 7 |
| Risk of bias in studies | 18 | Present assessments of risk of bias for each included study. | 7 |
| Results of individual studies | 19 | For all outcomes, present, for each study: (a) summary statistics for each group (where appropriate) and (b) an effect estimate and its precision (e.g. confidence/credible interval), ideally using structured tables or plots. | 7 |
| Results of syntheses | 20a | For each synthesis, briefly summarise the characteristics and risk of bias among contributing studies. | 7,8 |
|  | 20b | Present results of all statistical syntheses conducted. If meta-analysis was done, present for each the summary estimate and its precision (e.g. confidence/credible interval) and measures of statistical heterogeneity. If comparing groups, describe the direction of the effect. | 7,8 |
|  | 20c | Present results of all investigations of possible causes of heterogeneity among study results. | 7,8 |
|  | 20d | Present results of all sensitivity analyses conducted to assess the robustness of the synthesized results. | 7,8 |
| Reporting biases | 21 | Present assessments of risk of bias due to missing results (arising from reporting biases) for each synthesis assessed. | 7 |
| Certainty of evidence | 22 | Present assessments of certainty (or confidence) in the body of evidence for each outcome assessed. | 7,8 |
| **DISCUSSION** | | |  |
| Discussion | 23a | Provide a general interpretation of the results in the context of other evidence. | 8, 9,10,11 |
|  | 23b | Discuss any limitations of the evidence included in the review. | 10,11 |
|  | 23c | Discuss any limitations of the review processes used. | 10,11 |
|  | 23d | Discuss implications of the results for practice, policy, and future research. | 11 |
| **OTHER INFORMATION** | | |  |
| Registration and protocol | 24a | Provide registration information for the review, including register name and registration number, or state that the review was not registered. | 11,12 |
|  | 24b | Indicate where the review protocol can be accessed, or state that a protocol was not prepared. | 3 |
|  | 24c | Describe and explain any amendments to information provided at registration or in the protocol. | n/a |
| Support | 25 | Describe sources of financial or non-financial support for the review, and the role of the funders or sponsors in the review. | n/a |
| Competing interests | 26 | Declare any competing interests of review authors. | n/a |
| Availability of data, code and other materials | 27 | Report which of the following are publicly available and where they can be found: template data collection forms; data extracted from included studies; data used for all analyses; analytic code; any other materials used in the review. | n/a |

Appendix B. Search Strategy

| **Database** | **Specificities of the database** | **Search strategy** |
| --- | --- | --- |
| PubMed | All field | (Supplement or Caffeine or Creatine or Carbohydrate or Beetroot or Citrulline or Amino acids or Potassium Nitrate or Coffee or Beta-alanine or Taurine or Sodium Bicarbonate) AND (Postactivation or Postactivation Performance Enhancement) |
| Web of Sceience | Topic | (TS=(Supplement or Caffeine or Creatine or Carbohydrate or Beetroot or Citrulline or Amino acids or Potassium Nitrate or Coffee or Beta-alanine or Taurine or Sodium Bicarbonate)) AND TS=(Postactivation or Postactivation Performance Enhancement) |
| SPORTDiscus | Abstract/Title | (Postactivation or Postactivation Performance Enhancement or pap or pape or conditioning activity) AND (Supplement or Caffeine or Creatine or Carbohydrate or Beetroot or Citrulline or Amino acids or Potassium Nitrate or Coffee or Beta-alanine or Taurine or Sodium Bicarbonate) |

Appendix C. GRADE Assessment Specific Results

| Table. S1 GRADE assessment for the certainty of evidence | | | | | | | | | | |
| --- | --- | --- | --- | --- | --- | --- | --- | --- | --- | --- |
| Certainty assessment | | | | | |  | Effect size | | | Certainty |
| No. of studies | Risk of bias | Inconsistency | Indirectness | Imprecision | Publication  bias |  | No. of  participants | | Absolute (95% Crl) |  |
| *PAPE + Supplement vs. PAPE + PLA on performance outcomes* | | | | | | | | | | |
| 10 | not serious | not serious | not serious | Serious  -2 | none |  | 198 | | ES 0.78 (0.58 to 0.98) | ⨁⨁○○ Low |
| *PAPE + Supplement vs. PAPE + PLA on fatigue resistance outcomes* | | | | | |  |  |  |  |  |
| 4 | not serious | not serious | not serious | Serious  -2 | yes |  | 77 | | ES 0.72 (0.47 to 0.98) | ⨁○○○ Very low |
| *PAPE + Supplement vs. WU + Supplement on performance outcomes* | | | | | |  |  |  |  |  |
| 2 | not serious | not serious | not serious | Serious  -2 | yes |  | 34 | | ES 0.88 (0.58 to 1.18) | ⨁○○○ Very low |
| *PAPE + Supplement vs. WU + PLA on performance outcomes* | | | | | |  |  |  |  |  |
| 2 | not serious | not serious | not serious | Serious  -2 | yes |  | 40 | | ES 0.88 (0.58 to 1.18) | ⨁○○○ Very low |
| *PAPE + Supplement vs. WU on performance outcomes* | | | | | |  |  |  |  |  |
| 3 | not serious | serious | not serious | Serious  -2 | yes |  | 64 | | ES 0.88 (0.58 to 1.18) | ⨁○○○ Very low |
| **Note:** The *risk of bias* depends on the Rob-2 risk assessment results; *Inconsistency* depends on *I²* results, primarily whether the sum of between-studies and within-studies effects exceeds 50%; The *imprecision* primarily depends on whether the sample size exceeds 400 and whether the effect size exhibits a clear directionality (95% confidence interval crossing the zero point). | | | | | | | | | | |

Appendix D. Risk of publication bias and model diagnosis

**Figure S1.** Funnel plot and Egger's regression test. The effects of PAPE + supplement compared to PAPE + placebo on sports performance (a); The effects of PAPE + supplement compared to warm-up + supplement (b), warm-up + PLA (c), and warm-up alone (d) on sports performance.

**Figure S2.**  Loo elpd and Pareto k values. After removing the outliers, the model did not show any significant changes.

**Figure S3.**  Model diagnostic results.

**Figure S4.**  The effect of PAPE + supplementation compared to PAPE + placebo on fatigue resistance. (a) Model diagnostic results (b) Funnel plot and Egger regression test results (c) LOO elpd and Pareto k values.

**Figure S5.** Sensitivity analysis: Re-fit the model after removing values where Pareto k > 0.7. The effects of PAPE + supplement compared to PAPE + placebo on sports performance (a) and fatigue index (b); The effects of PAPE + supplement compared to warm-up + supplement (c), warm-up + PLA (d), and warm-up alone (e) on sports performance.

**Figure S6.** Sensitivity analysis. The effects of PAPE + supplement compared to PAPE + placebo on sports performance. (a) Variance–covariance estimation was not performed; (b), (c), (e), and (f) were modeled with pre–post correlation coefficients of 0.9, 0.8, 0.7, and 0.6, respectively, and variance–covariance estimation was also conducted; (d) was modeled using only post-test data, for which variance–covariance estimation was performed.

**Figure S7.** Sensitivity analysis. The effects of PAPE + supplement compared to PAPE + placebo on fatigue index. (a) Variance–covariance estimation was not performed; (b), (c), (e), and (f) were modeled with pre–post correlation coefficients of 0.9, 0.8, 0.7, and 0.6, respectively, and variance–covariance estimation was also conducted; (d) was modeled using only post-test data, for which variance–covariance estimation was performed.

**Figure S8.** Sensitivity analysis. The effects of PAPE + supplement compared to warm-up + supplement (a, b) and warm-up + PLA (c, d). (a) and (c) Variance–covariance estimation was not performed; (b) and (d) were modeled using only post-test data, for which variance–covariance estimation was performed.

**Figure S9.** Sensitivity analysis. The effects of PAPE + supplement compared to warm-up alone on sports performance. (a) Variance–covariance estimation was not performed; (b), (c), (e), and (f) were modeled with pre–post correlation coefficients of 0.9, 0.8, 0.7, and 0.6, respectively, and variance–covariance estimation was also conducted; (d) was modeled using only post-test data, for which variance–covariance estimation was performed.

Appendix E. Network meta-analysis.


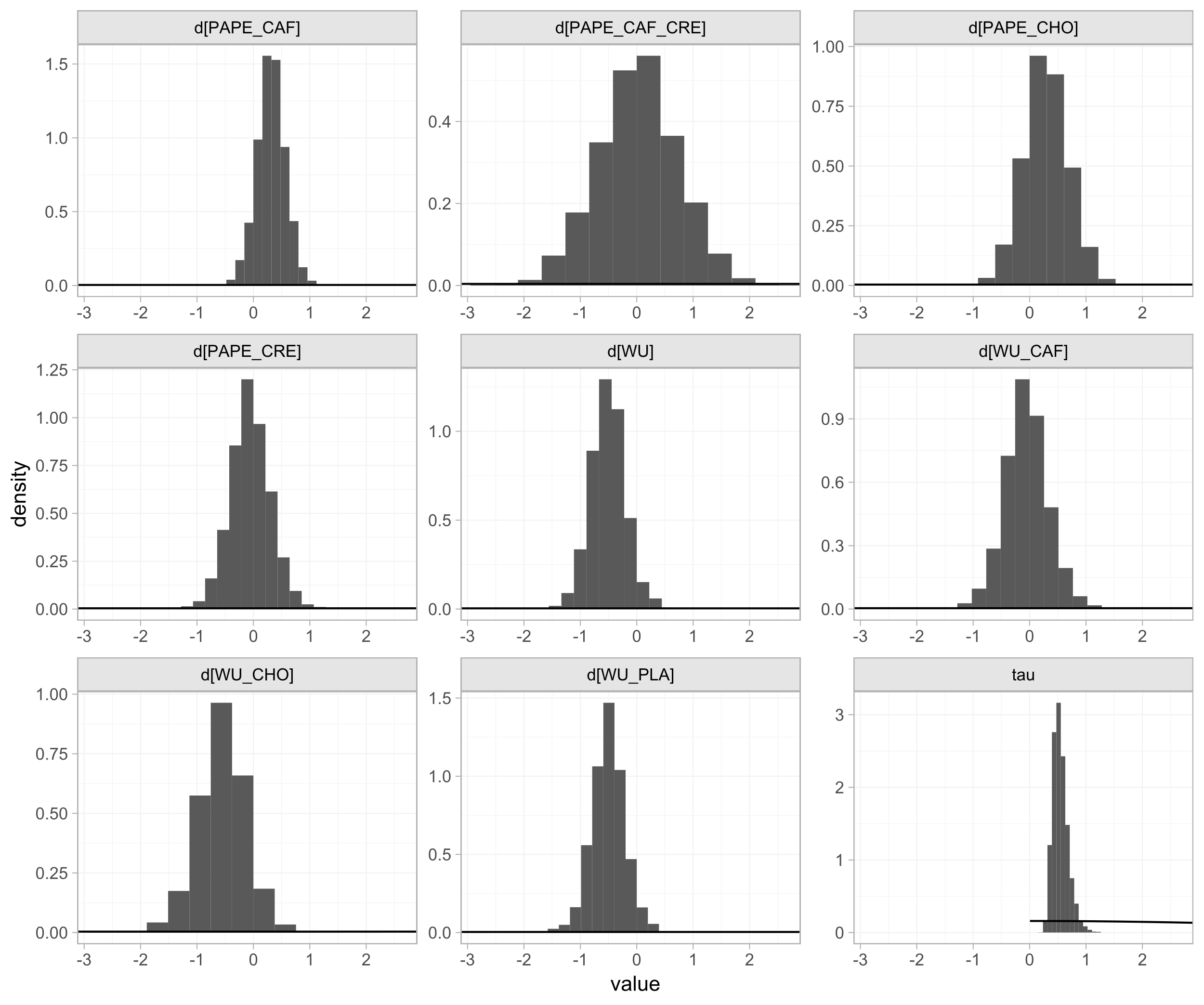


**Figure S10:** Posterior distribution after network meta-analysis.


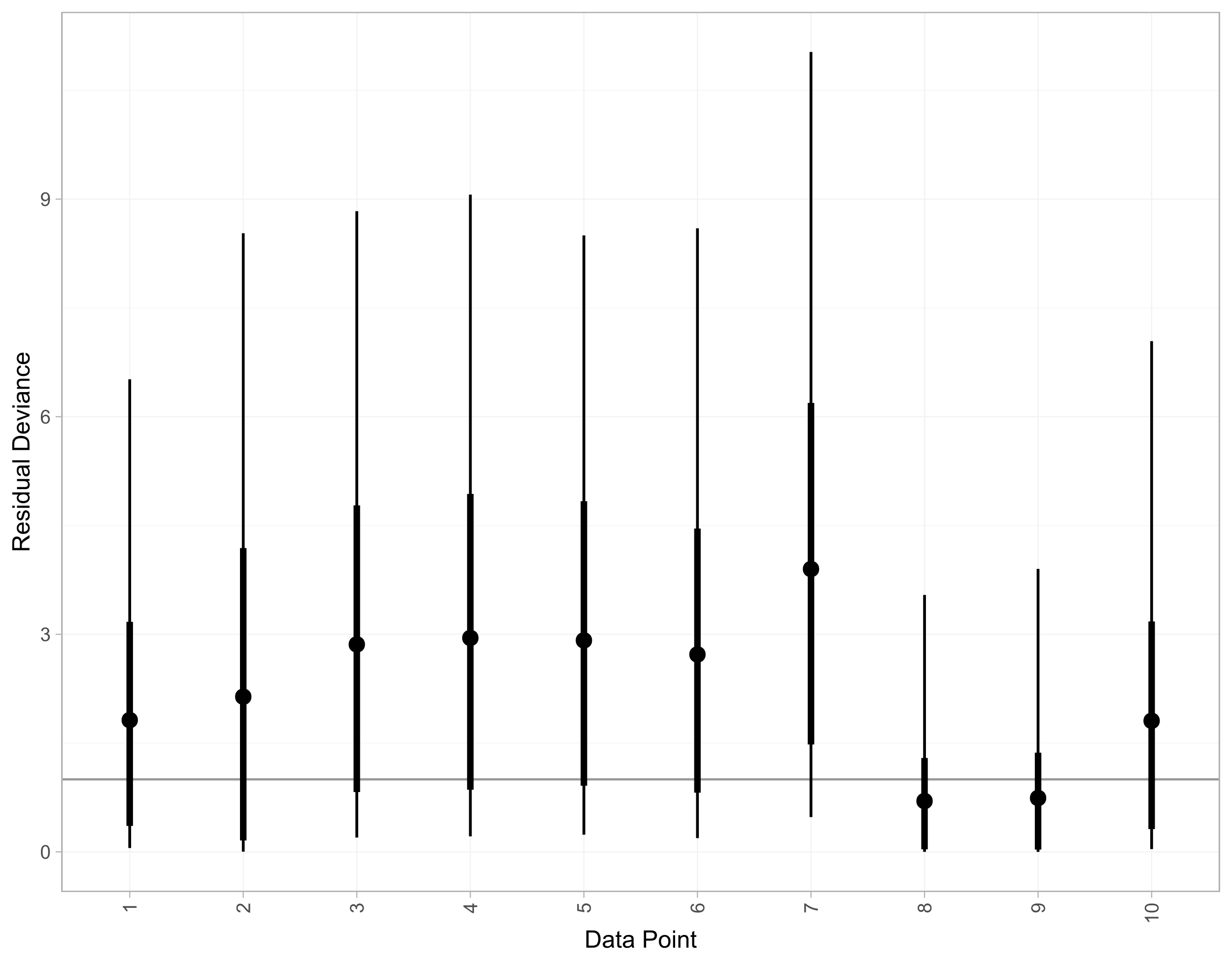


**Figure S11:** Residual Deviance plot.


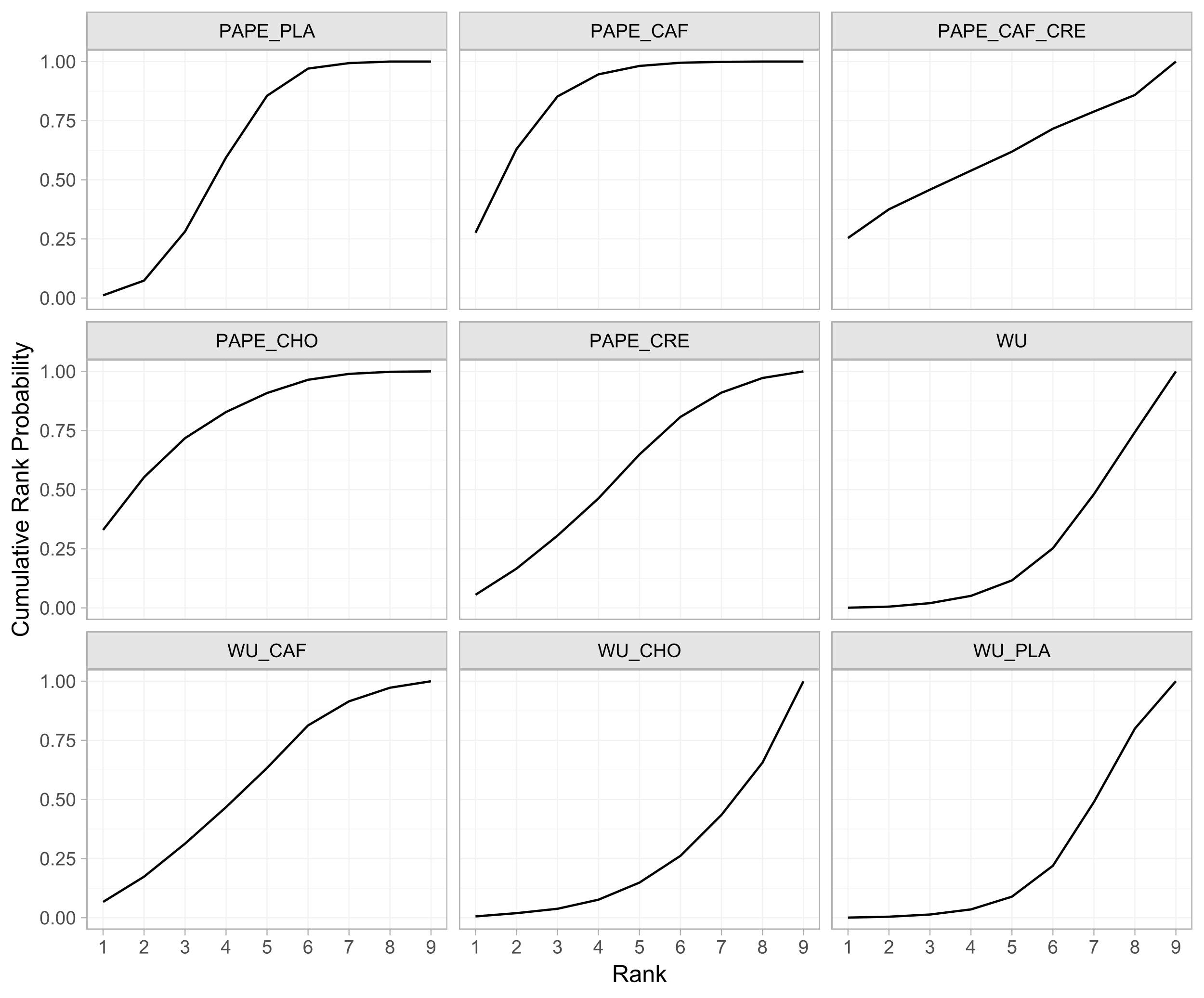


**Figure S12:** SURCA rank.
